# Supplementary material for: Total Cholesterol and Mortality in Older Adults: A Sex-Stratified Cohort Study
Source: Nutrients. 2025 Sep 30;17(19):3128. doi: 10.3390/nu17193128 (PMC12525712; doi:10.3390/nu17193128)
Supplement: Supplementary file 1 [file nutrients-17-03128-s001.zip › nutrients-3851177-supplementary.pdf]

Supplementary table S1. Adjusted cox proportional hazards models for all-cause mortality by sex and total cholesterol levels

Sex	Total cholesterol (mg/dl)	Adjusted HR[§]	95% CI[§]
Overall*	<200	-	-
	200-239	0.69	0.49-0.96
	≥240	0.73	0.49-1.08
**Men****	<200		
	200-239	1.05	0.67 - 1.63
	>=240	0.74	0.40 - 1.36
**Women****	<200		
	200-239	0.47	0.28- 0.77
	>=240	0.72	0.42 - 1.22

* *Adjusted for age, sex, physical activity, diabetes, hypertension, COPD, CKD-EPI, BMI, frailty, polypharmacy, statin use, thyroid disease, liver disease, alcohol consumption, and statin use.*
** *Adjusted for age, physical activity, diabetes, hypertension, COPD, CKD-EPI, BMI, frailty, and polypharmacy, statin use, thyroid disease, liver disease, alcohol consumption, and statin use.*
[§]Estimates are hazard ratios (HR) with 95% confidence intervals; reference category is TC <200 mg/dL

Supplementary Table S2. Adjusted Cox proportional hazards models for all-cause mortality by sex and total cholesterol categories, restricted to participants with follow-up ≥365 days (1-year lag analysis).

Sex	Total cholesterol (mg/dl)	Adjusted HR[§]	95% CI[§]
Overall*	<200	-	-
	200-239	0.76	0.55-1.05
	≥240	0.75	0.51-1.10
Men**	<200		
	200-239	1.07	0.69 - 1.66
	>=240	0.83	0.46 - 1.47
Women**	<200		
	200-239	0.54	0.33- 0.89
	>=240	0.67	0.39 - 1.13

** Adjusted for age, sex, physical activity, diabetes, hypertension, COPD, CKD-EPI, BMI, frailty, polypharmacy, statin use, thyroid disease, liver disease, alcohol consumption, and statin use.*

*** Adjusted for age, physical activity, diabetes, hypertension, COPD, CKD-EPI, BMI, frailty, and polypharmacy, statin use, thyroid disease, liver disease, alcohol consumption, and statin use.*

[§]Estimates are hazard ratios (HR) with 95% confidence intervals; reference category is TC <200 mg/dL

Supplementary Figure S1. Restricted cubic splines (Harrell's 5-knot scheme; 5th, 27.5th, 50th, 72.5th, 95th percentiles of TC) for the association between TC and all-cause mortality: overall (left panel), men (middle panel), women (righ panel).

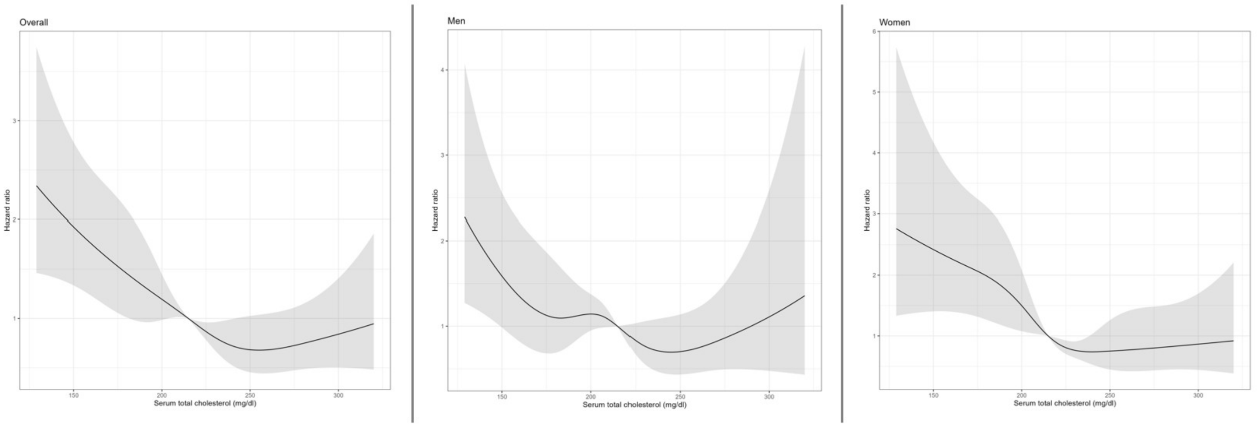

Solid line = adjusted HR; shaded area = 95% CI. HR is scaled to 1 at the reference TC.
